# Gut Microbiota – A Potential Contributor in the Pathogenesis of Bipolar Disorder

**DOI:** 10.3389/fnins.2022.830748

**Published:** 2022-03-23

**Authors:** Peifen Zhang, Lingzhuo Kong, Huimin Huang, Yanmeng Pan, Danhua Zhang, Jiajun Jiang, Yuting Shen, Caixi Xi, Jianbo Lai, Chee H. Ng, Shaohua Hu

**Affiliations:** ^1^Department of Psychiatry, The First Affiliated Hospital, Zhejiang University School of Medicine, Hangzhou, China; ^2^MOE Frontier Science Center for Brain Science and Brain-Machine Integration, Zhejiang University, Hangzhou, China; ^3^Department of Psychiatry, Wenzhou Medical University, Wenzhou, China; ^4^The Key Laboratory of Mental Disorder Management in Zhejiang Province, Hangzhou, China; ^5^Brain Research Institute, Zhejiang University, Hangzhou, China; ^6^Department of Psychiatry, The Melbourne Clinic and St Vincent’s Hospital, University of Melbourne, Richmond, VIC, Australia

**Keywords:** bipolar disorder, gut microbiota, gut-brain axis, pathogenesis, treatment

## Abstract

Bipolar disorder (BD) is one of the major psychiatric disorders that is characterized by recurrent episodes of depression and mania (or hypomania), leading to seriously adverse outcomes with unclear pathogenesis. There is an underlying relationship between bacterial communities residing in the gut and brain function, which together form the gut-brain axis (GBA). Recent studies have shown that changes in the gut microbiota have been observed in a large number of BD patients, so the axis may play a role in the pathogenesis of BD. This review summarizes briefly the relationship between the GBA and brain function, the composition and changes of gut microbiota in patients with BD, and further explores the potential role of GBA-related pathway in the pathogenesis of BD as well as the limitations in this field at present in order to provide new ideas for the future etiology research and drug development.

## Introduction

Bipolar disorder (BD) belongs to the subcategory of mood disorders, characterized by repeated episodes of major depression (lasting at least for 2 weeks) and mania (or hypomania), which subtypes include Bipolar type I and type II, the difference between which mainly lies in the severity of mania, and obvious differences in treatment and prognosis are also noticed. Late adolescence is the average age at which BD develops (Bauer et al., 2018). Epidemiological statistics showed that the prevalence of BD was about 2% (Merikangas et al., 2011), and the risk of mortality rate due to suicide, which partly attributed to the mood states of BD patients, was 20–30 times higher than that of the normal (Pompili et al., 2013; Orsolini et al., 2016; Fornaro et al., 2018; De Berardis et al., 2021). The early diagnosis of BD is difficult, and the final clinical diagnosis is usually made 5–10 years after the recurrence of the initial symptoms (Fortinguerra et al., 2019). Poor prognosis is another feature of BD, while cognitive dysfunction and poor social regression can be observed in many patients with chronic BD (Passos et al., 2016). However, the pathogenesis of BD has not been fully and clearly elucidated.

Enormous number of microbial communities in the intestinal tract form a symbiotic relationship with the host, contributing to the maintenance of homeostasis and potentially influencing the function of other organs (Sen et al., 2021), due to the ability of gut microbiota metabolites to act on the host neurotransmitter system (Ogawa et al., 2020), vagal reflex (Zhang et al., 2021) and immunoreaction (Haack et al., 2007). For example, the improvement of sleep quality (Marotta et al., 2019; Sen et al., 2021) can also be achieved through the application of intestinal microbiota or fecal flora transplantation. In addition, soluble CD14 (sCD14) in the serum indicates ectopic intestinal bacteria. The serum level of this marker was significantly increased in patients with a variety of diseases mainly characterized by brain dysfunction, such as schizophrenia, depression, and BD (Nguyen et al., 2018). These results suggest that the close relationship between intestinal microecology and brain function, which was called the “gut-brain” axis (GBA).

In fact, preliminary progress has been made on the link between intestinal microbiome homeostasis and the onset of BD. Karling et al. (2016) conducted a retrospective study of gastrointestinal symptoms in 136 patients with BD, and found a positive correlation between depressive symptoms and the occurrence rate of gastrointestinal symptoms. In a more recent study, Kao et al. (2019) found that patients with inflammatory bowel disease (IBD) were more likely to develop BD. This is in accordance with findings by Kostic et al. (2014). Although the above studies could not clarify the specific causal association, we can speculate that intestinal factors may play a certain role in the pathogenesis of BD.

In this review, we systematically review previous relevant studies and attempt to establish the association between the GBA and BD from the perspectives of neuroinflammation, molecular biology and neuropathology. We hope this work will provide evidence for future research in related fields.

## The Gut-Brain Axis: Why Choosing It?

It is well known that the human microbiome is vast. The microbes and their gene community together constitute the microbiome that maintains the physiological homeostasis of the organism, in which bacteria are the main component, and fungi, viruses and other mainly parasitic eukaryotic microorganisms are also included (Nguyen et al., 2018; Zhang et al., 2019). These microbes live in different parts of the body, including the skin, mouth and intestines (Costello et al., 2009). Colonization of gut microbes occurs during childbirth and is perfected during infancy (Dominguez-Bello et al., 2010). The importance of the microbiome has been gradually discovered through high-throughput sequencing techniques (Qin et al., 2010; Knight et al., 2017). The link between gut microbes and the development of the immune system was first proposed more than a decade ago (Round and Mazmanian, 2009). Subsequently, a number of studies have shown that changes in intestinal microbial composition and metabolic function can trigger a series of pathophysiological changes, such as activation of focal inflammatory responses and oxidative stress mechanisms (Kamada et al., 2013). Surprisingly, similar inflammatory changes were observed in psychiatric disorders represented by schizophrenia and BD (Kirkpatrick and Miller, 2013). These evidences link intestinal microorganisms with changes in physiological functions of the whole body, thus providing possible ideas for the etiological research of many diseases whose basic pathological mechanism is macro or micro inflammatory responses.

**FIGURE 1:**
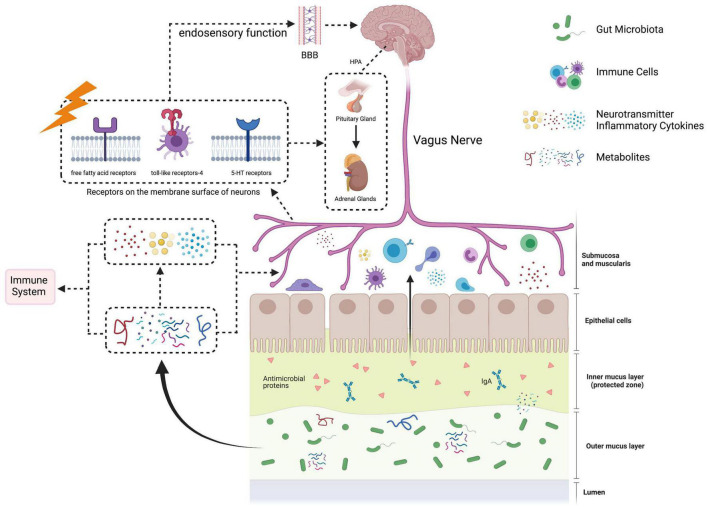
The paradigm of gut-brain axis. The gut-brain axis, referring to the signals’ bidirectional communication between gut microbiota and the brain, is mediated by neuronal, immune, and neuroendocrine pathways. In the gastrointestinal tract, certain gut microbiota can produce neuroactive substances, such as dopamine, serotine and short-chain fatty acids. Signals from abnormal gut microbiota and its metabolites can be sensed and transported by afferent fibers of vagus nerve into the brain. In addition, the integrity of gut barrier is disrupted to form the “leaky gut,” which facilitates gut microbiota-derived components and metabolites to enter the circulatory system. These substances can directly or indirectly activate the immune system to release inflammatory factors. Under the state of immune dysfunction, the permeability of the blood–brain barrier (BBB) is increased. Inflammatory factors and neuroactive substances produced by gut microbiota go through the BBB, leading to neuroinflammatory, activating the hypothalamus-pituitary-adrenal (HPA) axis and disrupting brain functions.

Theoretically, GBA contains multiple systems, i.e., nervous system, immune system, neuroendocrine system, etc. Microbial metabolism plays a role in initiating and communicating various systems in the GBA (Figure 1).

### Neuronal Pathways: The Mysterious Vagus

A growing number of studies suggest there may be a “potential” link between the brain and the gut. For example, Sherwin et al. (2019) have proposed that brain development is subject to microbiome changes – disturbances in the local microenvironment during the early maturation of the gut microbiome may lead to defects in neurogenesis, axon and dendrite growth, which may have an impact on early brain development, and even the entire life cycle of the body. What we need to know is that the gut microbiome, even though it can be thought of as a microbiome, is not uniform in time and space (Fulling et al., 2019). This results in differences in the composition of microbes in different parts of the gut, as well as metabolites, including sugars, short-chain fatty acids (SCFAs), and neuroactive substances such as serotonin (5-HT) and γ-aminobutyric acid (GABA) (Fulling et al., 2019). These metabolites can act on the local nervous system of the intestinal tract, of which the vagus nerve is the most widely studied.

Vagus nerve is the most important plant nerve controlling gastrointestinal function. Vagovagal reflex can inhibit gastric empty and promote the secretion of gastrointestinal glands, thus regulating the digestion and absorption function of the gastrointestinal tract. There are a large number of vagus nerve endings in the submucosa and muscularis of the intestinal mucosa, and these nerve endings account for about 90% of the total vagus nerve endings (Fulling et al., 2019). Meanwhile, the vagus nerve can also transmit information from the intestinal tract and other peripheral areas to the cerebral cortex, amygdala and hippocampus, thus playing a role in memory, emotion and cognition regulation (Wang et al., 2021). Bonaz et al. (2018) made an analysis on the vagus nerve and brain function, and they found that this plant nerve was capable of directly sensing neural activity signals from intestinal flora metabolites through the endosensory function of the afferent nerve and 5-HT receptors, toll-like receptors (TLR) 4, and free fatty acid receptors distributed on the surface of the vagus nerve, after which it sent afferent signals to the brain. After cutting the vagus nerve of the experimental animals, the researchers found that the effects of *Lactobacillus rhamnosus JB-1* against anxious behavior and elevated GABA receptor expression were reduced (Bravo et al., 2011). Additionally, nerve fiber connections between the gut vagus nerve and dopamine reward pathways in the striatum of the basal ganglia have been demonstrated in previous experiments (Han et al., 2018). Functional neuroimaging studies have shown that functional changes in the dopamine reward pathway may be the cause of some mental illnesses, such as substance addiction (Lindgren et al., 2018), mood disorders (Serafini et al., 2020), and eating disorders (Lindgren et al., 2018). We speculate that stimulation of the vagus nerve may be able to “awaken” the dopamine reward pathway to achieve intervention in these illnesses (Han et al., 2018).

### Immune System: Neuroinflammation and Immuno-Response

Neuroinflammation and immune responses have been observed in a variety of mental illnesses, such as depression (Troubat et al., 2021), BD (Benedetti et al., 2020), and schizophrenia (Buckley, 2019). In these common psychiatric disorders, the inflammatory response is mainly reflected in the activation of immune cells in the nervous system dominated by microglia, which leads to the release of a series of pro-inflammatory and anti-inflammatory cytokines (Kim and de Vellis, 2005; Beurel et al., 2020). Long-term chronic inflammation will change the microenvironment around neurons, leading to a series of adverse consequences, such as neuron damage, impaired cell signal transduction mechanism and neurotransmitter reuptake dysfunction (Stephan et al., 2012). These subtle changes exacerbated brain dysfunction. In short, inflammation is a double-edged sword. Failure to achieve appropriate balance can lead to unpredictable damage.

Neuroimmune response can be modulated by many variables, among which the role of intestinal microbes cannot be ignored. The role of intestinal microbes in immune regulation can be roughly divided into immune cells and cytokines. Intestinal microorganisms mainly regulate T-line immune cells, including Th-1, Th-2, Th-17, and Treg, etc. In an animal study of symbiotic regulation, Ivanov et al. (2009) found that mice colonized by *segmented filamentous bacterium* in the lamella propria of terminal ileum mucosa produced immune effector TH-17 cells and CD4 (+) helper T cells. The same results were found in the experiments of Lee et al. (2020). Meanwhile, mice fed with *Bacteroidetes fragilis* polysaccharides elicited a specific Th-1 cell response (Jakobsson et al., 2014), while *Bifidobacterium* inhibited the action of Th-2 cells (Fang et al., 2020). Other studies have found that the differentiation process of Treg cells is affected by a variety of factors. Differentiation of Treg cells was inhibited in the mesenteric lymph nodes of mice that carried *Akkermansia muciniphila* and *Acinetobacter calcoaceticus* (intestinal flora transplants from multiple sclerosis patients) in their intestines (Berer et al., 2017).

In the central nervous system (CNS), microglia play a similar role to that of macrophages in peripheral tissues, namely non-specific immune recognition and phagocytosis. Microglia have been found to make up about 5–10% of all brain cells (Kim and de Vellis, 2005). These cells can proliferate and differentiate under the action of various stimulus factors, and produce cytokines and chemokines, thus mediating neuroinflammation and immune response, participating in maintaining synaptic plasticity (Yirmiya et al., 2015), and playing a role in neuronal network regulation and damage repair (Lenz and Nelson, 2018). A variety of factors can affect microglia homeostasis. Erny et al. (2015) treated germ-free (GF) mice with SCFAs produced by microbial fermentation, and individuals originally deficient in microglia function showed partial restoration of microglia function, whereas those changes were not observed in individuals with SCFAs receptor gene (FFAR2) defects. It suggests that the symbiotic microbiome can play a regulatory role in the function of microglia, that is, the complexity of the effective microbiome can indirectly affect the immune status of CNS. This conclusion provides a scientific basis for establishing the GBA functional model.

The TLR is a series of cell receptors expressed in neurons, glial cells and non-specific immune cells, and plays an important role in the immune system’s recognition of bacterial lipopolysaccharides. Intestinal microbes activate TLR, which in turn promotes the release of a series of cytokines (e.g., IL-1, IL-6, TNF-α, etc.) that can cross the blood–brain barrier (BBB), thus affecting the function of CNS (Lucidi et al., 2021). BBB originally had a certain permeability to cytokines such as TNF-α. While in GF mice, this permeability is significantly increased, resulting in significantly elevated levels of cytokines in the cerebrospinal fluid (CSF), leading to a series of CNS inflammation, and ultimately lead to dysfunction of neural pathways (Braniste et al., 2014). In the peripheral immune system, various circulating cytokines can also be regulated by intestinal flora and its metabolites. The relative abundance of *Blautia* could affect plasma IFN-γ levels, and there was a negative correlation between them. With the increase of *Pseudomonas*, *Streptomyces*, *Clostridium* and *Bacillus* abundance, IFN-γ levels also increased (Cao et al., 2021).

### Neuroendocrine and Metabolic Pathways: Associated With Emotion

The regulation of emotion is influenced by many factors. Among these factors, neurotransmitters are the most widely studied. The role of GABA is mainly inhibitory, while glutamate and dopamine can promote the production of positive emotions, which has almost become a consensus in psychiatry. In previous studies of brain neurotransmitters, high concentrations of GABA in the synaptic cleft and decreased sensitivity of 5-HT receptor was associated with depression (depressive symptoms of BD, and other mood disorders as well) (Mahar et al., 2014). The abnormal release of dopamine can lead to mental disorders characterized by emotional overactivity, such as schizophrenia (Howes et al., 2017) and anxiety disorder (Sun et al., 2019). In addition, the excitatory toxicity mechanism of quinolinic acid which is reasonable for affecting synaptic plasticity has been found (Potter et al., 2010). Kynurenine can be further metabolized through the kynurenic acid and quinolinic acid pathways. Kynurenic acid has a neuroprotective effect against *N*-methyl-D-aspartate receptor antagonists, but high concentrations of quinolinic acid can lead to synaptic dysfunction, which is associated with cognitive deficits (Kadriu et al., 2019).

Gut microbes produce some neurotransmitters directly or indirectly. For example, *Bifidobacterium* and *Lactobacillus* are able to produce GABA (Patterson et al., 2019), which contributes to inhibitory changes in brain function. While *Lactobacillus*, *Oscillibacter*, and *Blautia* can promote the synthesis of 5-HT by increasing the expression level of tryptophan synthase gene (Busnelli et al., 2019; Chen et al., 2019). In fact, about 95% of 5-HT is derived from this synthetic pathway (Yano et al., 2015). Although we present the neurotransmitter here as a separate component, its changes are in fact the result of a combination of factors, including vagus nerve signaling stimulation, as well as inflammation and immune responses. Meanwhile, gut microbes can interfere with the body’s immune function. Some gut microbes (usually named “probiotics”) produce SCFAs (LeBlanc et al., 2017), which affect the immune function of the intestinal mucosa (Correa-Oliveira et al., 2016). Alternatively, activation of afferent signals in the vagus nerve via G-protein-coupled receptors (Nohr et al., 2015) or histone deacetylases (Waldecker et al., 2008) may affect the functions of macrophages, dendritic cells, monocytes and neutrophils, and affects the recruitment and differentiation of T cells (Correa-Oliveira et al., 2016). This pathway is mediated by immune cells and therefore ultimately comes down to changes in the body’s immune function. When the body’s immune function is impaired, the metabolism of kynurenine is accelerated, which changes the efficiency of GABA and dopamine secretion (Schwarcz and Stone, 2017). This is another way that the intestinal flora affects the levels of neurotransmitters in the brain.

## Gut-Brain Axis and Bipolar Disorder: Potential Connections

### Alterations of Gut Microbiota in Bipolar Disorder

**FIGURE 2:**
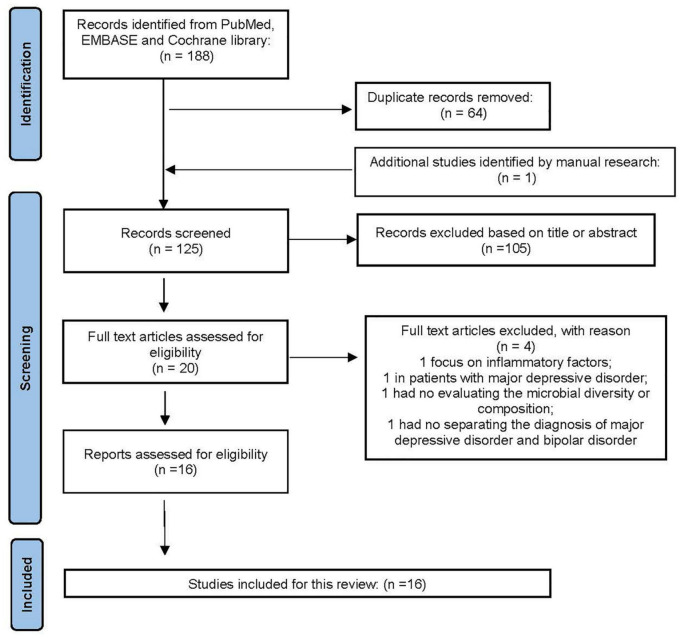
The PRISMA flow diagram.

As an important part of affective disorders, the relationship between BD and GBA has always been the focus of research. We summarized the existing studies and showed relevant research results in Table 1. The searched strategy in this review was consistent with the Preferred Reporting Items for Systematic Reviews and Meta-analyses (PRISMA). Relevant studies were searched via PubMed, EMBASE and Cochrane databases before December 01, 2021. The selected keywords were (“bipolar disorder” AND “gut microbiota”). We also searched the relevant articles, manually. Studies eligible for inclusion needed to be further filtered by human studies and original research papers, rather than reviews, letters, or meeting abstracts. Duplicate records were removed followed by titles and abstracts screening, as well as the full article. The process of this study selection is shown in Figure 2. Eventually, 16 articles on the gut microbiota in BD were included.

**TABLE 1:** Clinical studies on the relationship between gut microbiota and bipolar disorder (BD).

Country	Sample size	Gender	Mean age (years)	Sequence	Diversity/abundance	Association/conclusion	References
Denmark	• BD: 113 • Healthy first-degree relatives (HR): 39 • HCs: 77	• BD: 43M/70F • HR: 18M/21F • HCs: 30M/47F	–	16S rRNA	Alpha-diversity: NA. At the genus level: *Flavonifractor* ↑ in BD	*Flavonifractor* was associated with BD.	Coello et al., 2019
	• MZA: 7 • MZH: 32 • MZL: 25	• MZA: 18M/53F • MAH: 9M/23F • MZL: 5M/20F			Alpha-diversity: ↓ in MZA. The family *Christensenellaceae* ↓ in MZA and MZH.	A low relative sequence abundance of unclassified *Firmicutes* was associated with disease.	Vinberg et al., 2019
United States	• BD: 115 • HCs: 64	• BD: 32M/83F • HCs: 24M/40F	• BD: 50.2 (*SD*: 12.8) • HCs: 48.6 (*SD*: 16.6)		*Faecalibacterium* and unidentified member belongs to the R*uminococcaceae* family ↓ in BD	*Faecalibacterium* associated with better self-reported health outcomes including improved physical health, depression, and sleep quality scores. *Anaerostipe* and *Ruminococcaceae* associated with improved PCS while Enterobacteriaceae associated with worse PCS scores.	Evans et al., 2017
	• BD: 28 • SZ: 9	–			Diversity of *Alistipes*: ↓ in AAP-treated females	AAP treatment is associated with measurable differences in gut microbiota.	Flowers et al., 2019
	• BD treated with APP (APP): 49. • BD treated without APP (no-APP): 68.	• APP: 12M/37F. • No-APP: 48M/20F.	• APP: 51.7 (*SD*: 13.5). • No-APP: 46 (*SD*: 12)		The species diversity ↓ in AAP-treated females, while *Lachnospiraceae* ↑ in AAP-treated subjects.	AAP treatment associates with specific representation of gut bacterial families in patients receiving treatment.	Flowers et al., 2017
Austria	BD: 32 (Depression = 13; Euthymia = 19)	BD: 25M/7F	BD: 41.67 (*SD*: 17.51)		Alpha-diversity: ↓ in depression with BD.	Methylation of ARNTL correlates significantly with bacterial diversity and evenness in BD.	Bengesser et al., 2019
	• BD: 32. • HCs: 10.	• BD: 18M/14F • HCs: 4M/6F	• BD: 41.31 (*SD*: 14.73). • HCs: 31.4 (*SD*: 7.61)		Alpha-diversity: NA. Compared to HCs: the phylum *Actinobacteria*, class *Coriobacteriia* ↑ and Ruminococcaceae, *Faecalibacterium* ↓ in BD.	A negative correlation between microbial alpha-diversity and illness duration in BD. Bacterial clades associated with inflammatory status, serum lipids, TRP, depressive symptoms, oxidative stress, anthropometrics and metabolic syndrome in individuals with BD.	Painold et al., 2019
	BD: 20	BD: 11M/9F	BD: 51.50 (*SD*: 11.54)	–		A significant improvement of performance concerning attention and psychomotor processing speed measured was observed 1 month as well as 3 months treatment with probiotic supplements.	Reininghaus et al., 2018
China	• DA: 1 • BDB: 1 • HCs: 2 healthy spouses	• BDA: 1F • BDB: 1F • HCs: 2M/2F	**—**	16S rRNA	Compared to HCs: ACE and Chao ↓ in acute BD state. *Ruminococcaceae* and *Faecalibacterium* ↑ during the responsive and remissive BD states; *Enterobacter*↓ from the active state to remissive state of BD.	LPS biosynthesis genes were overrepresented in the gut microbiota of the active BD samples. Treatments with the potential microbiota might be helpful in BD.	(Jiang et al., 2019)
	• BD: 52 • HCs: 45	• BD: 27M/25F • HCs: 22M/23F	• BD: 24.15 (*SD*: 9.5) • HCs: 36.29 (*SD*: 12.22)		Alpha-diversity: ↓ in untreated BD. Compared to HCs: *Firmicutes phylum*, *Roseburia*, *Faecalibacterium*, *Coprococcus genera* ↓ *Bacteroidetes phylum*, *Parabacteroides*, *Bacteroides*, and *Halomonas genera* ↑ in untreated BD. After treatment with quetiapine: *Klebsiella* and *Veillonella* ↑ in treated BD.	MADRS scores were negatively correlated with the levels of *Acetanaero* bacterium, *Stenotrophomonas*, *Anaerotruncus*, and *Raoultella*, but positively correlated with *Acinetobacter* and *Cronobacter*. The duration of illness was positively correlated with *Allisonella* abundance, while negatively correlated with *Escherichia*/*Shigella, Flavonifractor*, *Staphylococcus* abundance.	Hu et al., 2019
	BD: 217; MDD: 165 HCs: 217	BD: 116M/101F MDD: 59M/106F HCs: 95M/122F	–		Compared to HCs: alpha-diversity: ↓ in BD. Compared with MDD: at the phylum levels: *Proteobacteria*↑ *while Bacteroidetes*↓ in BD. At the family levels: *Bacteroidaceae* and *Veillonellaceae*↓ while *Enterobacteriaceae* and *Pseudomonadaceae*↑ in BD.	26 differential OTUs is identified that can distinguish patients with MDD and BD. The family *Lachnospiraceae* were significantly associated with HAMD in MDD or BD patients.	Zheng et al., 2020
	BD: 30 MDD: 31 HCs: 30	BD: 15M/15F MDD: 9M/22F HCs: 14M/16F	BD: 38.40 (*SD*: 8.33) MDD: 41.58 (*SD*: 10.40) HCs: 39.47 (*SD*: 10.22)	SMS	Compared to HCs: Alpha-diversity: ↓ in MDD while NA in BD. The Gm coefficient ↓ in BD and MDD. At the phylum levels, *Firmicutes* and *Actinobacteria*↑ *Bacteroidetes*↓ and at the genus level, *Bacteroides*, *Clostridium*, *Bifidobacterium*, *Oscillibacter*, *Streptococcus* ↑ in MDD and BD.	HAMA positively correlated with Gm coefficient while negatively correlated with Shannon index only in BD group.	Rong et al., 2019
	BD: 25 HCs: 28	BD: 14M/11F HCs: 13M/15F	BD: 36.92 (*SD*: 10.14) HCs: 39.21 (*SD*: 11.11)		Compared to HCs: alpha-diversity: Fisher index ↓ while Shannon index ↑ in BD. Compared to HCs: at the phylum levels: *Actinobacteria* and *Firmicutes*↑ while *Bacteroidetes*↓ in BD.	Alterations of the microbes may have the potential as biomarkers for distinguishing the BD patients from the HCs. The potential function of the microbes may affect the normal operation of the TRP system in BD patients.	Lai et al., 2021
	• BD: 36 (BD-I: 10; BD-II: 26) • HCs: 27	• BD: 21M/15F • HCs: 15M/12F	• BD: 32.64 (*SD*: 10.643) • HCs: 28.89 (*SD*: 11.095)	qPCR	Compared to HCs: the ratio of B/E ↓ in BD. The counts of *Faecalibacterium prausnitzii, Bacteroides Prevotella* group, *Atopobium* cluster, *Enterobacter* spp., and *Clostridium* Cluster IV ↑ in BD.	The log_10_B-G_CB_ and log_10_ Enterobacter spp. count were positively correlated with the CD3^+^ T cell proportion.	Lu et al., 2019
Canada	• BD: 23 (BD-I: c15; BD-II: 8) • HCs: 23	• BD-I: 6M/9F • BD-II: 1M/7F • HCs: 7M/16F	• BD-I: 43.5 (*SD*: 17.25) • BD-II: 49 (*SD*: 10.5) • HCs: 43.75 (*SD*: 18.25)	PCR	Diversity: ↓ in BD. *Clostridiaceae* OUT and *Collinsella* ↑ in BD, while *Collinsella* ↑ in BD-II.	Individuals with BD may have a distinct gut microbiota profile compared to healthy controls, with a greater abundance of *Clostridiaceae* and *Collinsella*.	McIntyre et al., 2021
Japan	• BD: 39 (BD-I: 13; BD-II: 26) • HCs: 58	• BD: 17M/22F • HCs: 22M/36F	• BD: 40.3 (*SD*: 9.2) • HCs: 43.1 (*SD*: 12.9)	RT-qPCR	–	A negative correlation between *Lactobacillus* counts and sleep. A significant negative correlation was found between *Bifidobacterium* counts and cortisol levels.	Aizawa et al., 2018

*BD, bipolar disorder; HCs, healthy controls; NA, no difference; LPS, lipopolysaccharide; ACE, angiotensin converting enzyme; BDA, BD affected twin A; BDB, BD unaffected twin B; MZA, monozygotic (MZ) twins affected twins with unipolar or bipolar disorder in remission; MZH, unaffected monozygotic twin at familial risk as their co-twin had a diagnosis of affective disorder; MZL, no monozygotic twins or first-degree history of affective disorder; ARNTL, a clock gene that can be methylated; MADRS, Montgomery–Åsberg Depression Rating Scale; PCS, physical health. B/E, Bifidobacterium ratio Enterobacteriacea ratio and represents microbial colonization resistance; AAP, atypical antipsychotic; PCR, polymerase chain reaction; qPCR, quantitative polymerase chain reaction; RT-qPCR, real-time quantitative polymerase chain reaction; FMT, fecal microbiota transplantation; MDD, major depressive disorder; SMS, shotgun metagenomics sequencing. TRP, tryptophan.*

Alpha diversity is a comprehensive measure that assesses the richness and evenness of species. In general, the alpha diversity of intestinal flora in BD patients showed a decreasing trend (Hu et al., 2019). The alpha diversity of intestinal flora decreased with the increase of the methylation ratio of a clock gene, *ARNTL* (Bengesser et al., 2019). A longitudinal case study of twins with BD suggests that increased alpha diversity of gut flora may be associated with improved depressive symptoms (Vinberg et al., 2019). Generally speaking, changes in the composition of intestinal flora or metabolic capacity are associated with the severity of BD symptoms, the selection of therapeutic agents, and the evaluation of therapeutic effects. More specifically, changes in gut microbiota composition were found in patients with BD, and it is believed to be associated with the development of BD. A number of studies on BD and intestinal flora have found that the abundance of one of the butyrate-producing bacteria – *Faecalibacterium* – is observed to decrease in BD patients with acute mood changes, while the above indicators increase again when the mood is softened (Hu et al., 2019; Painold et al., 2019). Other butyric acid producing bacteria, such as *Roseburia* and *Coprococcus*, were also at low levels in BD patients, suggesting that the reduction of this beneficial bacteria may be the cause of the disorder (Hu et al., 2019). Lu et al. (2019) sampled and analyzed the intestinal flora of BD patients before and after quetiapine treatment, and found that the proportion of *Bifidobacteria* and *Enterobacteriaceae* increased. BD patients treated with probiotics showed a significant reduction in symptoms and improvement in cognitive function (Aizawa et al., 2018; Reininghaus et al., 2018).

### Pathways That the Gut-Brain Axis Mediates in Bipolar Disorder

Previous research results show that the pathogenesis of BD is complicated. Under the combined action of a variety of internal and external factors (such as genetic susceptibility, external environmental stimulation and homeostasis disorders, etc.), the characteristic clinical manifestations and auxiliary examination abnormalities of BD are finally formed. Among different proposed mechanisms for BD, the role of GBA is gradually recognized by many researchers, who believe that studies on the GBA-related pathways may help to clarify the pathogenesis of BD.

The BD is genetically hereditable. A paper published in 2018 showed that 85% of BD cases can be inherited in families (Vieta et al., 2018). Goodrich et al. (2014) suggested that this genetic trait might be related to the interaction between genetic variations and gut microbes. Genetic phenotypes cannot only affect the composition of intestinal microbes, but also affect the metabolic pattern of the body through microbes. This raises a whole new question – can genetic traits link gut microbes to BD? A genome-wide association study was conducted to evaluate the gene-microbiome correlation in BD patients, and the results showed that the increased level of *Desulfovibrio* community was associated with the genetic characteristics of the host, and both showed a significant correlation with BD (Cheng et al., 2020). In addition, in a study of BD patients with identical twins, Jiang et al. (2019) found that compared with healthy partners, the intestinal microbiota spectrum of BD patients with identical twins was more different. After complete remission of BD, this difference was significantly reduced. Thus, we can speculate that gut microbes play a role in the formation of BD phenotypes.

The conjecture about the connection between vagus nerve and brain function has been explained above. Generally speaking, we need to pay attention to the relationship between the vagus nerve and the intestinal flora, and the effects of the vagus nerve on the onset of BD. There is increasing evidence that the vagus nerve may be a “bridge” between GBA and brain function. Based on this theory, a new type of physical therapy has emerged in recent years. Vagus nerve stimulation – the use of electrodes to stimulate the vagus nerve in the neck – was initially used to control acute seizures and shorten the duration of drug treatment. Now, this surgery has achieved some remarkable results in improving the clinical symptoms of BD patients (McAllister-Williams et al., 2020). In addition, a preclinical study showed that the depressive-like phenotype in experimental mice was related to an overactivation of the vagus nerve (Wang et al., 2020). The gut microbiota of the mice was artificially altered by a diet containing *Lactobacillus intestinalis* and *Lactobacillus reuteri*. However, despite these evidences, the specific relationship between vagus nerve and GBA remains to be confirmed by further experiments.

As for the pathophysiological mechanism of BD, existing studies mainly focus on the neurotransmitter imbalance (Newberg et al., 2008). The formation and regulation of emotion and cognitive function is complicated and tedious. The homeostasis of excitatory and inhibitory neurotransmitters and their interactions all play an important role. Previous studies have suggested that the dopamine system (mesencephalon-limbic system, mesencephalon-cortical system, substantia nigra-striatum system, and nodular-funnel system) and reward circuits may be involved in the manic symptoms of schizophrenia and BD (Berk et al., 2007), while executive function relies on the GABA neurotransmitter (Huber et al., 2018). Meanwhile, studies on BD also show that an improvement of the efficiency in 5-HT transmission is beneficial to alleviate the severity and duration of BD depressive symptoms (Ananth et al., 2020). As mentioned above, the production of these neurotransmitters also depends on intestinal microbes. Therefore, intervention in the composition or metabolic function of intestinal microbes may become a new approach for the treatment of mental diseases represented by BD. In addition to the common neurotransmitters, some neuroactive substances produced by gut microbes can influence the concentration or delivery efficiency of neurotransmitters. For example, kynurenine is produced by the fermentation of tryptophan, a neuroactive substance that inhibits 5-HT synthesis (O’Mahony et al., 2015). Hydroxykynurenine is neurotoxic, and accumulation of this substance may lead to neurotoxic reactions that intervene neurotransmitter function (Bartoli et al., 2020).

The GBA contains multiple pathways with potential for interaction. For example, short-chain fatty acids can alter neurotransmitter levels, activate the vagus nerve, and trigger an immune response via activation of T cells and macrophages (Dalile et al., 2019). The metabolism of kynurenine is influenced by immune function, and abnormal metabolism can affect the secretion of neurotransmitters such as dopamine and GABA (Schwarcz and Stone, 2017). In summary, the occurrence of BD is closely related to the GBA, which is in line with the multi-factor hypothesis of the pathogenesis of BD, namely, functional abnormalities of the monoamine neurotransmitter system and immune system, and changes in connections between neurons (Vieta E). Given the lack of current research results, it is still a new way to clarify the pathogenesis of BD from the GBA perspective and develop new anti-BD drugs that interfere with intestinal microorganisms.

## Therapies Connecting Gut Microbiata and Bipolar Disorder

In view of the high prevalence of BD, traditional treatment methods for BD include mood stabilizers (carbamazepine, lithium carbonate, etc.), atypical antipsychotics (quetiapine, olanzapine, etc.), traditional antidepressants (SSRIs, SNRIs, etc.), psychotherapy, and peer support (McCormick et al., 2015). Although existing treatment methods and clinical care are more or less effective for not a few patients, the prognosis and the social burden caused by BD still should not be underestimated (Dilsaver, 2011). Management of BD patients is a major challenge for healthcare professionals. One reason for this result is that there is still a significant proportion of patients who have not achieved complete remission and functional recovery. These patients may experience relapse or aggravation of the disease, lack of social support, adverse effects of medications and other adverse outcomes directly or indirectly caused by BD. Given the association between the GBA and BD, we speculate that regulation of gut microbiome may be an attractive and promising treatment for BD.

### Probiotics and Prebiotics

Probiotics are defined as beneficial living bacteria, and prebiotics contain non-digestible fibers which can enhance the probiotics’ function. Particularly, some probiotics are called “psychobiotics,” and the term refers to their effects on mental illnesses (Barbosa and Vieira-Coelho, 2020). Animal studies indicated that administration of probiotics could upregulate neuronal and synaptic connections and exert anti-depressive effects (Capuco et al., 2020). A clinical study showed that the severity of both depression and mania was reduced after 8 weeks’ probiotic supplement in patients with type I BD, which proved the benefits showed by that kind of “psychobiotics” (Eslami Shahrbabaki et al., 2020). Dickerson et al. (2018) reported remission in manic symptoms and reduction in rehospitalization rate after treatment with *Bifidobacterium* and *Lactobacillus*, especially in BD patients with manic episodes and high baseline levels of systemic inflammation. Cognitive impairments may persist in euthymic periods of BD and thus result in low quality of life. Reininghaus et al. (2018) found that the attention and executive functions were enhanced after 3-month treatment with probiotic in 20 individuals with euthymic BD, indicating that probiotics supplement could improve the cognition dysfunctions. These preliminary data support that prebiotics and probiotics were beneficial to regulate the mood and behaviors and may be promising treatment strategies for BD. In spite of these findings, more efforts are needed before the clinical application of psychobiotics because the proper dosages, duration and adverse effects are still needing of robust evidence.

### Diet

As one of the bodily systems interacting with foods, manipulation of gut microbiota via dietary adjustment has garnered much attention. Mediterranean diet (MD) emphasizes a balanced consumption of fruits, vegetables, legumes, unsaturated fats with limited red meat intake rather than excluding any particular or specific food. MD could enhance the abundance of *Prevotella* and SCFAs producing bacteria such as *Faecalibacterium* and *Clostridium* whereas decrease the phylum *Bacteroides* (Jin et al., 2019). Researches have shown that MD was correlated with improvement of cognitive functions and emerged as an accessible strategy to manage depressive syndromes (Nagpal et al., 2019). Dietary interventions enriched in omega-3 polyunsaturated fatty acids can improve the abundance of beneficial bacteria, such as *Bifidobacterium* and *Lactobacillus*, and dampen the HPA-axis activity in mice (Robertson et al., 2017). Mice fed with a high-fat diet elevated the *Firmicutes*-*Bacteroidetes* ratio and displayed anxiety-like behaviors (Marx et al., 2021). In general, dietary patterns are considered as influential factors in shaping gut microbiota and present as potential treatment targets in mediating brain health.

### Exercise

Regular exercise offers a strategy to regulate physiological responses including the inhabiting of gut microbiota. Compared to individuals who always keep sedentary, the diversity of microbiota in professional athletes was elevated and the profile of beneficial metabolites especially SCFAs was enriched (Barton et al., 2018). In addition to the altered β diversity of gut microbiota, Allen et al. (2018) also found that SCFAs were increased through exercise intervention, especially in individuals with a lean body. In marathon athletes, increased abundance was observed in *Veillonella atypica*, which is a genus that can metabolize lactate into propionate, a sort of SCFAs (Scheiman et al., 2019). At present, SCFAs have been regarded as a mediator in the gut and brain due to their neuroactive, anti-inflammatory role and beneficial effects on brain health. Therefore, we can speculate that exercise can also be an approach to modulate the composition and metabolic capacity of gut microbiota in BD individuals.

### Fecal Microbiota Transplantation

Fecal microbiota transplantation (FMT), a direct method to manipulate the microbiota, refers to transfer the stool samples from a donor to a recipient, in order to observe effects caused by the factors lying in the stool of the donor (Dabke et al., 2019). In patients with *Clostridium difficile* infection, FMT has been proved to exert therapeutic effects through shaping the gut microbiota (Hvas et al., 2019). Up to now, there has been one clinical study depicting FMT in BD. A woman diagnosed with BD went through FMT from her healthy husband at least nine times. Interestingly, she became free from manic or depressive symptoms in the sixth month and had a weight loss of nearly 33 kg (Hinton, 2020). These positive results provide hope that FMT would be an emerging therapy for abnormal neurologic and behavioral conditions, though many difficulties and issues affecting donors and recipients still exist.

In summary, preliminary progress has been made in the treatment of BD based on intestinal microbiome and the GBA. However, due to the uncertainty of the specific mechanism of the axis, new therapeutic methods are still in the stage of exploration. Encouragingly, a growing number of clinical experimental therapies are showing advantages over traditional BD therapies, such as the avoidance of adverse drug reactions, significantly shorter response times, and improved patient compliance. It suggests that with the continuous clarification of various mechanisms in the GBA pathway, the treatment based on intestinal microbiome may replace the traditional treatments in the future, becoming an alternative choice for BD patients.

## Discussion

There are many causes leading to BD via a complex interaction of various factors. Abundant experimental evidence suggests that elucidating the mechanism of GBA will help to explain the etiology of BD. There is an obvious correlation between the changes of intestinal microbiome and the pathogenesis and clinical manifestations of BD – truly a breakthrough, since it may open up a new idea for the diagnosis and management of BD.

This review focuses on the possible mechanism of GBA, etiology and susceptibility factors of BD, and summarizes recent research conclusions on the relationship between the GBA and BD. Through the combination of sequencing technology and bioinformatics analysis, it is very important to identify the functional genes of specific microorganisms and their encoding enzymes involved in metabolic pathways, which is the fundamental work in this field. At present, the most surprising discovery lies in that, on the one hand, the composition of intestinal microbe or metabolic function of BD patients is different from that of healthy individuals, and these changes are more obvious in acute mood episodes (Painold et al., 2019; Vinberg et al., 2019). On the other hand, artificially altering the gut microbiome (e.g., using probiotics, or changing the composition of the feed) of subjects or laboratory animals can lead to secondary changes in brain functions (Wang et al., 2020). However, these observational results do not determine any change that is specially linked to certain microbe, since individual heterogeneity cannot be ruled out. Under different living habits and demographic background, the composition ratio of intestinal flora varies among different individuals. In addition, there are still many microorganisms whose metabolic mechanisms have not been thoroughly studied, so conclusions cannot be drawn arbitrarily.

Based on the above conclusions, some treatments for BD specific to the GBA have been found. Compared to traditional treatments, these new treatments focus on gut microbes, which are treated in a gentler and more patient-friendly way, through probiotics, diet, exercise and FMT. This, to some extent, alleviates the problem of reduced patient compliance caused by adverse reactions to psychotropic drugs. In fact, traditional BD medications such as mood stabilizers, antidepressants, and antipsychotics are “symptomatic treatment” rather than “causal treatment,” This is because most of the mechanisms of action of these drugs involve direct intervention of brain function via the neurotransmitter system – which is why these drugs (especially mood stabilizers and antipsychotics) are commonly used for the treatment of acute mood episodes. The treatment based on intestinal microbiome is worthy of explorations, because there has been more and more evidence suggesting that the disorder of intestinal microenvironment is one of the causes of BD.

Certainly, studies of the relationship between gut microbiota in BD patients are still at a juvenile stage up to now. Considering the cross-sectional research shortcoming, the knowledge gap about the dynamic alterations of gut microbiota in BD patients need to be filled through longitudinal cohorts. When it comes to the impact factors on gut microbiota, and medication is not able to be neglected. However, most individuals are currently treated with medication prior to recruitment. Thus, the randomized controlled trials would be undertaken to eliminate confounding factors. At the same time, the complex of gut microbiota is obvious. In addition to bacteria, the intestinal microbiome also contains fungi, virus, and bacteriophages. The relationship among gut microbiota and metabolomes, peptidomes, transcriptomics and brain function, etc., is yet unknown. Therefore, the combined analysis of multi-omics provides greater insight into gut microbiota framework. Despite lack of animal models close to humans, pre-clinical experiments are still crucial to identify the specific mechanism of gut microbiota in BD.

## Conclusion

The BD is a mental illness caused by a variety of factors. It is characterized by hidden early symptoms, long course of disease and poor prognosis in severe cases. Research on the etiology of BD has always been a hot issue in psychiatry. In recent years, important advances have been made in the biological role of gut microbiota in BD. Existing experiments provide evidence that one of the etiologies of BD is the disturbance of intestinal ecosystem, and the structural basis of the association between BD and the GBA. The periodic fluctuation of BD patients’ mood is believed to be related to gut microbiota, which produces a series of metabolites that can affect brain function through vagus nerve, immune system and other pathways. Therefore, treatment targeting gut microbiota may be a potential adjunctive therapy for BD. But the research is still in its infancy, and the available evidence is still insufficient for clinical application. It is necessary to further determine the types of gut microbiota and the specific mechanism of action in BD, and develop more feasible treatment methods in the future.

## Author Contributions

PZ and SH: conceptualization. PZ and LK: writing—original draft preparation. LK: visualization. HH, YP, DZ, JJ, CX, and YS: literature. JL, CN, and SH: writing—review and editing. JL and SH: funding acquisition. All authors have read and agreed to the published version of the manuscript.

## Conflict of Interest

The authors declare that the research was conducted in the absence of any commercial or financial relationships that could be construed as a potential conflict of interest.

## Publisher’s Note

All claims expressed in this article are solely those of the authors and do not necessarily represent those of their affiliated organizations, or those of the publisher, the editors and the reviewers. Any product that may be evaluated in this article, or claim that may be made by its manufacturer, is not guaranteed or endorsed by the publisher.
